# Adaptation and validation of the Ugandan Primary Care Assessment Tool

**DOI:** 10.4102/phcfm.v15i1.3835

**Published:** 2023-01-19

**Authors:** Innocent K. Besigye, Robert Mash

**Affiliations:** 1 Department of Family and Emergency Medicine, Faculty of Medicine and Health Sciences, Stellenbosch University, Cape Town, South Africa; 2 Department of Family Medicine, School of Medicine, Makerere University, Kampala, Uganda

**Keywords:** primary health care, primary care, Primary Care Assessment Tool, primary health care performance, coordination, comprehensiveness, continuity, person-centredness

## Abstract

**Background:**

Health systems based on primary health care (PHC) have better outcomes at lower cost. Such health systems need regular performance assessment for quality improvement and maintenance. In many low- and middle-income countries (LMICs), there are no electronic databases for routine monitoring. There is an urgent need for valid and reliable tools to measure PHC performance.

**Aim:**

This study aimed to adapt and validate the Primary Care Assessment Tool (PCAT) in the Ugandan context.

**Setting:**

The experts that participated in the Delphi process were recruited from almost all over the country.

**Methods:**

The study utilised a Delphi process with a panel of 20 experts (14 district health officers, 4 academics in primary care and 2 ministry of health [MOH] technical staff) who responded to iterative rounds of questionnaires in order to reach consensus (defined as > 70% agreement).

**Results:**

Consensus was reached after two rounds of the Delphi. In round one, four items in the comprehensiveness domain (services available) were removed and five items needed rephrasing. A new domain on person-centredness with 13 items was suggested. In round two, the new domain with each and every single one of its items and the items for rephrasing all achieved consensus. The final Ugandan version of the PCAT (UG-PCAT) has 12 domains and 91 items.

**Conclusion:**

The South African Primary Care Assessment Tool (ZA PCAT) was adapted and validated with an additional domain on person-centredness to measure primary care performance in the Ugandan context, and can now be used to measure the quality of core functions of primary care in Uganda.

**Contribution:**

The PCAT could fulfil the need for such a tool in a wider LMIC context. The UG-PCAT will be used to measure the quality of these core functions in Uganda and to assist with the improvement of PHC.

## Introduction

Health systems based on primary health care (PHC), with well-established and functioning primary care, have better health outcomes at lower cost.^1,2,3^ The declaration of Alma Ata in 1978 was the first global consensus to articulate the role of PHC in health systems with an ambitious goal of ‘Achieving Health for All’ by the year 2000.^4^ Although many global, regional and national stakeholders have attempted to promote and implement PHC, there is still significant inequity in health, with almost half of the world’s population having no access to high quality PHC.^5^ As a result, there is a high prevalence of unaddressed individual and population health needs in many parts of the world.

The World Health Report 2008, ‘PHC Now More than Ever’, emphasised people-centred health services that are responsive to the needs of people and communities.^6^ The 2018 Declaration of Astana re-affirmed the pivotal role of PHC in achieving Universal Health Coverage (UHC) and health-related Sustainable Developmental Goals (SDGs).^7^ In order to achieve the aspirations of the Declaration of Astana, the World Health Organization (WHO) published an operational framework for PHC as a guide to achieve high performing PHC systems.^8,9^ Strong PHC systems must have high quality primary care, whose performance should be continuously monitored for improvement. Therefore, valid and reliable tools are needed to measure primary care performance to identify performance gaps which can then be targeted for improvement.

The global need to measure the performance of PHC has led to the development and validation of several tools and frameworks, which include the Primary Care Assessment Tool (PCAT),^10^ the Quality of Outcomes Framework (QOF),^11^ the Primary Health Care Performance Initiative (PHCPI) framework,^12^ the European Primary Care Monitoring Framework (EPCMF),^13^ and the Patient Centred Primary Care Measure (PCPCM).^14^ The latest global approach to PHC performance measurement is the WHO framework embedded within the PHC theory of change as described in the PHC operational framework.^15^ These tools and frameworks have been used to measure PHC performance in different countries around the world.^16,17^ Such tools should effectively measure the core functions of primary care through patient surveys as recommended by WHO.^18^ The core functions of primary care are defined as first-contact accessibility, comprehensiveness, continuity, coordination, and person-centredness. Among all these tools and frameworks, the PCAT has been widely used to measure PHC performance focusing on the core functions as experienced by patients.

There is need for robust evidence to guide primary care policy and resource allocation, particularly in low- and middle-income countries (LMICs) struggling with weak health systems. Most studies on PHC have focused on policy, payment systems, workforce, community engagement, frameworks for performance management, provider competence, provider motivation, provider-patient relationships, and person-centredness as well as comprehensiveness of care.^19^ Most measurement indicators focus on inputs and outputs, particularly coverage and population health for prioritised conditions such as human immunodeficiency virus (HIV), tuberculosis (TB), malaria and maternal mortality, with relative neglect of the broader system, service delivery and final outcomes.^20^ Primary health care is a complex system and there is no single tool that can measure all the domains included in its framework. Different tools have been developed to measure particular components of PHC, and the PCAT measures the core functions of primary care.^15^

Countries with well-established health information systems and large electronic databases can more easily monitor the performance of their primary care systems. This is not so easy in LMICs, where there are often no electronic medical records and minimal routinely collected electronic data. In LMICs, routine health information systems have been implemented to collect health information at regular intervals.^21^ Studies have found poor data quality, given the diverse methods and tools used in its collection.^22^ Therefore, data may be difficult to rely on as a basis for decision and policy-making. Additionally, such health information is collected with negligible, or no involvement of the people served. In measuring the quality of health services, particularly primary care, the key functions should be assessed from the patients’ perspective, and the PCAT tool allows such an approach.^10^

The PCAT has been used in several regions of the world to assess the quality of PHC services and some core primary care functions.^17^ However, the PCAT needs to be adapted to the local context for validity and reliability given the cultural and contextual differences that exist among populations and health systems around the globe. It is also important that tools to measure PHC performance are regularly updated and aligned with new ideas, policies and guidelines by global bodies such as the WHO. This study aimed to adapt and validate the South African version of the PCAT to the Ugandan primary care context for use in measuring primary care performance in a district health system (DHS).

## Research methods and design

### Study design

The Delphi technique, with a panel of experts, was used to adapt and validate the South African Primary Care Assessment Tool (ZA PCAT) for the Ugandan context.

### Setting

In Uganda, primary care is provided through the DHS that is composed of the Village Health Team (VHT) at level I, followed by different levels of Health Centres (from levels II to IV), all operating under the Health Sub-District (HSD) leadership and governance. The general hospital then forms the apex of the DHS for these primary care facilities. The Ministry of Health (MOH) formulates PHC policies and provides stewardship on their implementation and overall functioning of the health system. Primary care providers include nurses, midwives, dispensers, clinical officers (mid-level clinicians) and non-specialist doctors referred to as medical officers. Due to the scarcity of doctors, primary care services are mainly provided by clinical officers, nurses and midwives, with support from community health workers. This primary care system lacks gatekeeping and therefore is characterised by patients bypassing the lower-level health facilities to seek care in hospitals and specialised centres.

### Selection of the expert panel

A panel of 30 experts was purposively selected as recommended in literature.^23^ The selection was based on their conceptual understanding of primary care as well as the Ugandan context. Experts included family physicians (because of their specialist training in primary care), district health officers (because they oversee the district primary care system), primary care academics (because they are familiar with updates and trends in primary care), and technical personnel in the directorate of clinical services in the MOH (because they are familiar with primary care policy formulation and implementation). The selected experts were contacted by telephone and invited to participate in the study. All the contacted experts agreed to participate in the panel. The final panel included 10 family physicians, 14 district health officers, 4 academics and 2 technical staff from the MOH.

### The South African Primary Care Assessment Tool

The South African version of the PCAT (ZA PCAT) measured 11 primary care domains (Table 1) with 82 items. The tool had an additional three domains on the extent of affiliation to the primary care facility (6 items), self-assessment of overall health status (2 items), and respondents’ socio-demographic characteristics (12 items). Each item in the main domains was scored on a 4-point Likert scale (4 – definitely, 3 – probably, 2 – probably not and 1 – definitely not). Each item also has an additional option of ‘not sure/don’t remember’.

**TABLE 1:** Definitions of the Primary Care Assessment Tool domains.

Domain	Definition	Number of items
First contact (utilisation)	Utilisation of primary care services when a need for care arises. First contact refers to the primary care services being responsible for assisting the person in need of care to enter the health care system for each non-referred provision of healthcare.	3
First contact (access)	Care is first sought from accessible primary care services when a new health or medical problem arises. Primary care serves as the usual entry into the health care system.	5
On-going care	Longitudinal use of a regular source of care over time regardless of the presence or absence of disease or injury. A health care home is then established where the person seeks continuous care building a long-term relationship with the provider as well as fostering mutual understanding and knowledge of each other’s expectations and needs.	9
Coordination (health system)	Linking of health care visits and services so that patients receive care for all their health problems, physical as well as mental. Primary care systems taking responsibility and obligation to transfer information to and receive it from other sources that may be involved in the care of the patient.	10
Coordination (information system)	Availability of mechanisms to communicate information and use of that information in the person’s care plan.	3
Comprehensiveness (services available)	Availability of a wide range of essential health services in primary care that promote and preserve people’s health as well as providing care for illness and disability.	23
Comprehensiveness (services provided)	Appropriate provision of health care and essential health services in primary care across the entire spectrum that promote and preserve people’s health as well as providing care for illness and disability.	9
Family-centredness	Recognition of a family as a major participant in the assessment and management of the patient. Family-centred primary care recognises and incorporates knowledge of the family context (resources, risk factors and social factors) into the planning and delivery of primary care services.	3
Community orientation	Care that recognises the primary care needs of defined population. The effective delivery of services to individuals and communities is based on an understanding of their needs and the integration of their perspectives in the provision of health care. Primary care providers contribute to and participate in community assessment, health surveillance, monitoring and evaluation.	6
Cultural competence	Health care that respects the beliefs, interpersonal relationships, attitudes and behaviours of people and their influence on health. Services are designed to be acceptable to people distinguished by common values, language, heritage, and beliefs about health and disease within the communities served. The views of these groups should be determined and incorporated into decisions involving policies, priorities and plans related to the delivery of health care services.	5
PHC team	Availability of members of a multi-disciplinary PHC team such as social workers, therapists or community health workers.	6
Health assessment	Personal perception of one’s own health status.	2
Socio-demographic characteristics	Socio-demographic profile of the respondents.	12

*Source:* Definitions are according to the primary care theoretical framework and evaluating the performance of South African primary care study. Centre TJHPCP. The concept of primary care [homepage on the Internet]. Baltimore, MD: The Johns Hopkins Primary Care Policy Centre. 2022 [cited 2022 Aug 22]. Available from: https://www.jhsph.edu/research/centers-and-institutes/johns-hopkins-primary-care-policy-center/pca_tools.html#:~:text=The%20Primary%20Care%20Assessment%20Tools%20are%20appropriate%20for%20measuring%20the,key%20domains%20of%20primary%20care; Bresick G, Von Pressentin KB, Mash R. Evaluating the performance of South African primary care: A cross-sectional descriptive survey. S Afr Fam Pract. 2019;61(3):109–116. https://doi.org/10.1080/20786190.2019.1596666.^24,25^

PHC, primary health care.

### Consensus definition and achievement

Consensus was pre-defined as 70% agreement among the experts and four rounds were planned as follows:

***Round 1:*** The questionnaire was sent to the panel by email and a follow up telephone call prompted them to complete it. For each item, two questions were asked: (1) whether the topic addressed in the item was relevant to the Ugandan context and therefore should be kept, and if not relevant, then the experts were asked to explain why and suggest an equivalent replacement item, and (2) whether relevant items were phrased appropriately and if not, to suggest alternative wording. Experts were also asked to confirm the relevance of the domains and to suggest any new domains or items.

***Round 2:*** The items for which consensus was not achieved in round 1, together with any new suggested domains or items, and any suggested rephrasing of items were compiled into a new questionnaire. This questionnaire was again emailed to the panel. Any qualitative feedback and percentage scores for the items from round 1 were also included. The experts were asked to provide further feedback on these items. At the end of each section, the experts were again asked to give any additional qualitative feedback and to suggest any new domains and/or items relevant to the Ugandan context.

***Round 3:*** This was planned in the same way as round 2.

***Round 4:*** A face-to-face workshop was planned for the panel to reach a final decision on any items that did not achieve consensus in rounds 1–3. The nominal group technique (NGT) was identified as a useful process that could be used in the workshop.

### Ethical considerations

This study was approved by the Health Research Ethics Committee at Stellenbosch and the Makerere University School of Medicine Research and Ethics Committee. Written informed consent was sought from the experts before their participation in the Delphi process. The experts were informed that their participation was voluntary and that they can withdraw their participation at any stage of the process with no negative consequences. S20/04/103 and REC REF 2020-164. 26 May 2020 and 15 September 2020.

## Results

The final panel that actually engaged with the Delphi process consisted of seven family physicians, seven district health officers, four primary care academics, and two MOH technical staff in the directorate of clinical services. All 20 experts completed two rounds of the Delphi process, after which consensus was achieved on all domains and items.

### Round 1

The consensus of the panel was to retain all 11 domains. Within these domains, consensus for retention was achieved for 73 items. There was consensus to remove the following four items from the domain of comprehensiveness (services available):

- Checking to see if anyone in your family qualifies for any social grants, for example, old age pension, child support grant, disability, TB.
- Suggestions for nursing home care for someone in your family.
- Help with food supplements such as Ensure or food parcels.
- Access to termination of pregnancy services at or via your facility, if required.

Eight items required re-phrasing from the socio-demographic characteristics, comprehensiveness, and cultural competence domains (Table 2).

**TABLE 2:** Results of round 1 consensus process.

Section	Domain	Items presented	Consensus to retain	Consensus to remove	Items requiring re-phrasing
A	Extent of affiliation to primary care person or place	6	6	0	0
B	First contact-utilisation	3	3	0	0
C	First contact-access	5	5	0	0
D	On-going care	9	9	0	0
E	coordination (system)	10	10	0	0
F	Coordination (information systems)	3	3	0	0
G	Comprehensiveness (services available)	23	17	4	2
H	Comprehensiveness (services provided)	9	7	0	2
I	Family-centredness	3	3	0	0
J	Community-orientation	6	6	0	0
K	Cultural competence	5	4	0	1
P	Primary health care team	6	6	0	0
M	Health assessment	2	2	0	0
N	Demographic and socio-economic characteristics	12	9	0	3

Two expert panel members suggested an additional new domain ‘person-centredness’ and also suggested 13 items for the new domain based on a literature.^26^ All the re-phrased items and the items suggested for the new domain were included in a new questionnaire for round 2 of the Delphi.

### Round 2

Table 3 and Table 4 show the results of round 2. Table 3 shows items that were rephrased and on which the panel reached consensus in round 2.

**TABLE 3:** The rephrased items and rationale for rephrasing.

Original item	Rephrased item	Rationale for rephrasing the item
PAP smear tests for cervical cancer	Screening for cervical cancer.	Other methods such as visual inspection with acetic acid are used for screening.
Tests for cancer of the bowel, for example, examining the back passage	Testing for cancer of the bowel, for example, examining the anus or rectum	The words anus or rectum are better understood in the local context
Home safety, like storing medicines safely; safe use of paraffin stoves; gun safety; pesticides	Home safety, for example, storing medicines safely; safe use of paraffin stoves; use of pesticides	Gun safety was removed because very few homes have guns in Uganda
For females: how to prevent osteoporosis (i.e. softening of the bones); breast examination	For females: breast examination for cancer	Osteoporosis prevention is rarely considered as the population is skewed towards younger age groups
Would you recommend your CHC to someone who uses traditional medicine or home remedies such as Dutch medicines or herbs, or has special beliefs about health care?	Would you recommend your facility to someone who uses traditional medicine or home remedies?	There is no Dutch medicine in Uganda and special beliefs about health care would be difficult to define.
What is your home language?	What is your local language?	Local language is a more appropriate wording for the Ugandan context.
What is the highest grade that you completed at school?	What is the highest education level you completed at school?	The change was done to cater for the nomenclature of education grading in Uganda
Which of the following best describes your dwelling?	Which of the following best describes your home?	This was rephrased to match the easily understood word ‘home’.

PAP, Papanicolaou; CHC, community health centre.

**TABLE 4:** The added domain of person-centredness and its items.

Number	Item
**When you visit this facility, do health professionals usually:**	
Q1	Greet you in a way that makes you feel comfortable?
Q2	Encourage you to express your thoughts concerning your health problems?
Q3	Listen carefully to what you have to say?
Q4	Understand what you have to say?
Q5	Check to be sure they have understood everything?
Q6	Give you as much information as you want?
Q7	Check to see if the treatment plan is acceptable to you?
Q8	Spend the right amount of time with you?
Q9	Involve you in decisions about your health as much as you want?
Q10	Respond to your questions and concerns?
Q11	Encourage you to ask questions?
Q12	Discuss your reason(s) for coming to the facility?
Q13	Overall, are you satisfied with your visits to the healthcare workers at this facility?

*Source:* Wachira J, Middlestadt S, Reece M, Peng C-YJ, Braitstein P. Psychometric assessment of a physician-patient communication behaviors scale: The perspective of adult HIV patients in Kenya. AIDS Res Treat. 2013;2013:706191. https://doi.org/10.1155/2013/706191

The new domain person-centredness with all its items achieved consensus when presented to the panel of experts (Table 4). The 3rd and 4th rounds of Delphi were not done because all domains and items achieved consensus for inclusion in rounds 1 and 2. The final Ugandan version of PCAT (UG-PCAT) (published as a supplement to this article) has 12 domains and 91 items.

## Discussion

The UG-PCAT was adapted and validated through a 2-round Delphi despite the originally planned four rounds and an NGT. Four items were removed, six items were re-phrased, and a new domain on person-centredness with 13 items was added. The UG-PCAT is therefore very similar to the ZA-PCAT, and this may be a reflection of the broad similarities in context. The removed items, in the domain of comprehensive services available, reflect a different model of care, which does not include such services. This is because of the differences in approach to social services and legality of termination of pregnancy. Eight items were rephrased to suit the Ugandan vocabulary, culture and context.

Termination of pregnancy is illegal in Uganda and is only done for medical indications when three senior doctors agree.^27^ As one doctor must be a gynaecologist, this decision cannot be made in primary care settings. The Ugandan human resource policy allows employment of gynaecologists in secondary and tertiary care hospitals. Social grants, food supplementation and food parcels are not offered in the Ugandan setting. Social grants were a new phenomenon with the coronavirus disease 2019 (COVID-19) crisis, where certain vulnerable groups were offered food and money during lockdown.^28^ Care for patients in nursing homes is not possible as such homes are non-existent in both public and private settings. In adapting the tool, there is a tension between removing items that are not contextually relevant and removing items that may be considered essential to the measurement of that domain from a global PHC perspective. In our view, the removal of these items should not significantly weaken the reliability of the measurement of comprehensiveness in our context.

Adaptation and validation of PCAT tools has previously been done using panels of experts comprising primary care practitioners, family physicians, policymakers, health system managers, and in some cases health consumer organisations and patients’ representatives.^29,30,31^ This same approach was used with the experts assessing the content validity of domains and items rather than the construct validity. This approach of using multiple stakeholders caters for all perspectives within the complexity of primary care delivery. The process has usually involved a series of Delphi rounds with some including the NGT to achieve final consensus.^30,31^ This was not necessary in this study, maybe because the process in South Africa had already adapted the PCAT into the African context. Some authors have also utilised focus groups particularly for the rephrasing of items.^31^

The Kenyan version (KE-PCAT), also adapted from the ZA PCAT, is similar to the UG-PCAT with only three items removed and two rephrased.^32^ The same three items removed from the Kenyan version were also removed from the UG-PCAT and were from the same domain of comprehensiveness (services available). Therefore, other than the added person centredness domain to the UG-PCAT, the two versions are very similar. This helps to confirm the validity of the tool and the changes made for the East African context.

The original PCAT and the ZA-PCAT measure the core primary care functions of first contact access, continuity, comprehensiveness and coordination, but not person-centredness. Addition of the person-centredness domain to the UG-PCAT allows it to measure all the core functions as recently defined by the WHO.^18^ All the 13 items to measure the person-centredness domain were adopted from the Physician-Provider Communication Behaviours scale that was developed in neighbouring Kenya.^26^ All the items in the person-centredness domain achieved consensus for inclusion in only one round of the Delphi. Therefore, they are likely to be valid for the Ugandan context, which is very similar to the Kenyan one. However, they may need adaptation and validation in other African settings before use.

The UG-PCAT is quite lengthy compared to other versions and requires significant time to complete. This may compromise the feasibility of its use, particularly in busy primary care settings. However, it may also be possible to exclude the domains on teamwork, cultural competence, family-orientation and community-orientation, if the tool is streamlined to measure only the core functions of primary care as per the WHO measurement framework.^18^ The UG-PCAT therefore provides exactly the kind of tool envisaged by the WHO for measuring the core functions of primary care.

The process of adaptation and validation of the ZA-PCAT in Malawi involved exploratory factor analysis (EFA) with significant exclusion of both domains and items.^30^ Only 29 items remained in the Malawian version of the PCAT. Such reduction of domains and items makes the tool easy and quick to use, but also carries a high risk of losing the psychometric properties of the original version. Performing EFA on a tool that was already reliably constructed was probably unnecessary and resulted in a version that would be difficult to use outside of Malawi. The results of the Malawian PCAT will also be difficult to compare to results from the South African, Kenyan and Ugandan versions.

### Limitations

It is important to note that the PCAT does not necessarily measure what is important to the patients or users of health services, but their perception of the quality of primary care services. Therefore, other tools will be required to be responsive to patient satisfaction with services.

### Recommendations and implications

The UG-PCAT can now be used to measure the core functions of primary care in Uganda. The researchers plan to implement this within the rural Tororo district and investigate whether the measurements assist the district health services to improve quality of service delivery. Other researchers in the Ugandan context can also use the UG-PCAT.

Although all the domains were retained in this version of the UG-PCAT, it makes sense to focus on the domains that measure the core primary care functions as defined by the new WHO measurement framework.^18^ This will align the tool with the WHO framework and make it an attractive option for governments and health services to use in the African context. At the same time, this will also shorten the tool and make it more feasible to use.

Thought should be given to the creation of a PCAT tool that can be used across multiple countries in the region or even for sub-Saharan Africa as a whole. The South African, Kenyan and Ugandan versions of the PCAT are sufficiently similar to enable this.

Other versions of the PCAT (the managers’ and providers’ versions) should also be adapted and validated for use in the Ugandan context. These will help to comprehensively measure the performance of primary care from the perspectives of other key stakeholders in the delivery of quality primary care.

## Conclusion

The users’ version of the ZA-PCAT was adapted and validated to measure primary care performance in the Ugandan context. A new domain on person-centredness was added. The UG-PCAT is now able to measure the core functions of primary care as per the new WHO measurement framework. The PCAT could fulfil the need for such a tool in a wider LMIC context. The UG-PCAT will be used to measure the quality of these core functions in Uganda and to assist with the improvement of PHC.

## References

[1] Macinko J, Starfield B, Shi L. The contribution of primary care systems to health outcomes within Organization for Economic Cooperation and Development (OECD) countries, 1970–1998. Health Serv Res. 2003;38(3):831–865. 10.1111/1475-6773.00149 12822915 PMC1360919

[2] Shi L. The relationship between primary care and life chances. J Health Care Poor Underserved. 1992;3(2):321–335. 10.1353/hpu.2010.0460 1420668

[3] Franks P, Fiscella K. Primary care physicians and specialists as personal physicians. Health care expenditures and mortality experience. J Fam Pract. 1998;47(2):105–109. 9722797

[4] World Health Organization. Declaration of Alma-Ata [homepage on the Internet]. Geneva: WHO. 1978 [cited 2022 Jan 20]. Available from: https://www.who.int/publications/almaata_declaration_en.pdf

[5] World Health Organization. The International Bank for R, Development/The World B. Tracking universal health coverage: 2017 global monitoring report: Executive summary. Geneva: World Health Organization; 2017.

[6] Van Lerberghe W. The world health report 2008: Primary health care: Now more than ever. Geneva: World Health Organization; 2008.

[7] World Health Organization. Declaration of Astana [homepage on the Internet]. 2018 [cited 2022 Feb 07]. Available from: https://www.who.int/docs/default-source/primary-health/declaration/gcphc-declaration.pdf

[8] World Health Organization. A vision for primary health care in the 21st century [homepage on the Internet]. 2018 [cited 2022 Feb 07]. Available from: https://www.who.int/docs/default-source/primary-health/vision.pdf

[9] World Health Organization. Operational framework for primary health care: Transforming vision into action. Geneva: World Health Organisation; 2020.

[10] Shi L, Starfield B, Xu J. Validating the adult Primary Care Assessment Tool. J Fam Pract. 2001;50(2):161. 10.1037/t77102-000

[11] Roland M. Linking physicians’ pay to the quality of care – A major experiment in the United Kingdom. N Engl J Med. 2004;351(14):1448–1454. 10.1056/NEJMhpr041294 15459308

[12] Bitton A, Ratcliffe HL, Veillard JH, et al. Primary health care as a foundation for strengthening health systems in low- and middle-income countries. J Gen Intern Med. 2017;32(5):566–571. 10.1007/s11606-016-3898-5 27943038 PMC5400754

[13] Kringos DS, Boerma WG, Hutchinson A, Saltman RB, World Health Organization. Building primary care in a changing Europe. World Health Organization. Regional Office for Europe; 2015. 29035488

[14] Etz RS, Zyzanski SJ, Gonzalez MM, Reves SR, O’Neal JP, Stange KC. A new comprehensive measure of high-value aspects of primary care. Ann Fam Med. 2019;17(3):221–230. 10.1370/afm.2393 31085526 PMC6827628

[15] World Health Organization. Operational framework for primary health care [homepage on the Internet]. Geneva: WHO; 2020 [cited 2022 Aug 19]. Available from: https://www.who.int/publications/i/item/9789240017832

[16] Bresick GF, Sayed A-R, Le Grange C, Bhagwan S, Manga N, Hellenberg D. Western Cape Primary Care Assessment Tool (PCAT) study: Measuring primary care organisation and performance in the Western Cape Province, South Africa (2013). Afr J Prim Health Care Fam Med. 2016;8(1):e1–e12. 10.4102/phcfm.v8i1.1057 PMC4913443 27247157

[17] D’Avila OP, Pinto LF, Hauser L, Gonçalves MR, Harzheim E. The use of the Primary Care Assessment Tool (PCAT): An integrative review and proposed update. Cien Saude Colet. 2017;22(3):855–865. 10.1590/1413-81232017223.03312016 28300993

[18] World Health Organization. Primary health care measurement framework and indicators: Monitoring health systems through a primary health care lens [homepage on the Internet]. Geneva: WHO; 2022 [cited 2022 Mar 25]. Available from: https://www.who.int/publications/i/item/9789240044210

[19] Bitton A, Fifield J, Ratcliffe H, et al. Primary healthcare system performance in low-income and middle-income countries: A scoping review of the evidence from 2010 to 2017. BMJ Global Health. 2019;4(Suppl 8):e001551. 10.1136/bmjgh-2019-001551 PMC6703296 31478028

[20] Lanzara C, Stewart E, Gutierrez D, Hatt L. Taking stock of the global primary health care measurement landscape. 2020.

[21] Hotchkiss DR, Diana ML, Fleischman Foreit KG. How can routine health information systems improve health systems functioning in low- and middle-income countries? Assessing the evidence base. In: Menachemi N, Singh S, editors. Health information technology in the international context. Advances in health care management. Vol. 12. Bingley: Emerald Group Publishing Limited, 2012; p. 25–58. 10.1108/s1474-8231(2012)0000012006 22894044

[22] Hung YW, Hoxha K, Irwin BR, Law MR, Grépin KA. Using routine health information data for research in low- and middle-income countries: A systematic review. BMC Health Serv Res. 2020;20(1):790. 10.1186/s12913-020-05660-1 32843033 PMC7446185

[23] De Villiers MR, De Villiers PJT, Kent AP. The Delphi technique in health sciences education research. Med Teach. 2005;27(7):639–643. 10.1080/13611260500069947 16332558

[24] Centre TJHPCP. The concept of primary care [homepage on the Internet]. Baltimore, MD: The Johns Hopkins Primary Care Policy Centre. 2022 [cited 2022 Aug 22]. Available from: https://www.jhsph.edu/research/centers-and-institutes/johns-hopkins-primary-care-policy-center/pca_tools.html#:~:text=The%20Primary%20Care%20Assessment%20Tools%20are%20appropriate%20for%20measuring%20the,key%20domains%20of%20primary%20care

[25] Bresick G, Von Pressentin KB, Mash R. Evaluating the performance of South African primary care: A cross-sectional descriptive survey. S Afr Fam Pract. 2019;61(3):109–116. 10.1080/20786190.2019.1596666

[26] Wachira J, Middlestadt S, Reece M, Peng C-YJ, Braitstein P. Psychometric assessment of a physician-patient communication behaviors scale: The perspective of adult HIV patients in Kenya. AIDS Res Treat. 2013;2013:706191. 10.1155/2013/706191 23476754 PMC3582093

[27] Moore AM, Kibombo R, Cats-Baril D. Ugandan opinion-leaders’ knowledge and perceptions of unsafe abortion. Health Pol Plan. 2013;29(7):893–901. 10.1093/heapol/czt070 24064047

[28] Nathan I, Benon M. COVID-19 relief food distribution: Impact and lessons for Uganda. Pan Afr Med J. 2020;35(Suppl 2):142. 10.11604/pamj.supp.2020.35.2.24214 PMC7608770 33193957

[29] Wang W, Haggerty J. Development of Primary Care Assessment Tool-adult version in Tibet: Implication for low- and middle-income countries. Prim Health Care Res Dev. 2019;20:e94. 10.1017/S1463423619000239 32800017 PMC6609993

[30] Dullie L, Meland E, Hetlevik Ø, Mildestvedt T, Gjesdal S. Development and validation of a Malawian version of the Primary Care Assessment Tool. BMC Fam Pract. 2018;19(1):63. 10.1186/s12875-018-0763-0 29769022 PMC5956555

[31] Bresick G, Sayed A-R, Le Grange C, Bhagwan S, Manga N. Adaptation and cross-cultural validation of the United States Primary Care Assessment Tool (expanded version) for use in South Africa. Afr J Prim Health Care Fam Med. 2015;7(1):e1–e11. 10.4102/phcfm.v7i1.783 PMC4656921 26245610

[32] Mohamoud G, Mash R. The quality of primary care performance in private sector facilities in Nairobi, Kenya: A cross-sectional descriptive survey. BMC Prim Care. 2022;23(1):120. 10.1186/s12875-022-01700-3 35585488 PMC9114290

